# The impact of central obesity on the risk of hospitalization or death due to heart failure in type 1 diabetes: a 16-year cohort study

**DOI:** 10.1186/s12933-021-01340-4

**Published:** 2021-07-27

**Authors:** Erika B. Parente, Valma Harjutsalo, Carol Forsblom, Per-Henrik Groop, on behalf of The FinnDiane Study Group

**Affiliations:** 1 Folkhälsan Institute of Genetics, Folkhälsan Research Center, Helsinki, Finland; 2 Research Program for Clinical and Molecular Metabolism, Faculty of Medicine, University of Helsinki, Helsinki, Finland; 3 Department of Nephrology, University of Helsinki and Helsinki University Hospital, Helsinki, Finland; 4 National Institute for Health and Welfare, Helsinki, Finland; 5 Department of Diabetes, Central Clinical School, Monash University, Melbourne, VIC Australia

**Keywords:** Heart failure, Central obesity, Waist-height ratio, Type 1 diabetes, Nephropathy

## Abstract

**Background:**

Obesity and type 2 diabetes are well-known risk factors for heart failure (HF). Although obesity has increased in type 1 diabetes, studies regarding HF in this population are scarce. Therefore, we investigated the impact of body fat distribution on the risk of HF hospitalization or death in adults with type 1 diabetes at different stages of diabetic nephropathy (DN).

**Methods:**

From 5401 adults with type 1 diabetes in the Finnish Diabetic Nephropathy Study, 4668 were included in this analysis. The outcome was HF hospitalization or death identified from the Finnish Care Register for Health Care or the Causes of Death Register until the end of 2017. DN was based on urinary albumin excretion rate. A body mass index (BMI)  ≥  30 kg/m^2^ defined general obesity, whilst WHtR  ≥  0.5 central obesity. Multivariable Cox regression was used to explore the associations between central obesity, general obesity and the outcome. Then, subgroup analyses were performed by DN stages. Z statistic was used for ranking the association.

**Results:**

During a median follow-up of 16.4 (IQR 12.4–18.5) years, 323 incident cases occurred. From 308 hospitalizations due to HF, 35 resulted in death. Further 15 deaths occurred without previous hospitalization. The WHtR showed a stronger association with the outcome [HR  1.51, 95% CI (1.26–1.81), z  =  4.40] than BMI [HR 1.05, 95% CI (1.01–1.08), z = 2.71]. HbA_1c_ [HR 1.35, 95% CI (1.24–1.46), z = 7.19] was the most relevant modifiable risk factor for the outcome whereas WHtR was the third. Individuals with microalbuminuria but no central obesity had a similar risk of the outcome as those with normoalbuminuria. General obesity was associated with the outcome only at the macroalbuminuria stage.

**Conclusions:**

Central obesity associates with an increased risk of heart failure hospitalization or death in adults with type 1 diabetes, and WHtR may be a clinically useful screening tool.

**Supplementary Information:**

The online version contains supplementary material available at 10.1186/s12933-021-01340-4.

## Background

Diabetes is a major risk factor for heart failure (HF), furthermore, individuals with HF and diabetes have a worse prognosis than those without diabetes [1, 2]. However, HF is still underdiagnosed in individuals with diabetes [3–5] and sometimes the diagnosis occurs at hospital admission. The in-hospital and after-discharge mortality rate due to HF is considerably high [6]. Hence, finding predictors of HF in individuals with diabetes is warranted, especially if they are modifiable risk factors.

Obesity is a well-known risk factor for HF in the general population [2, 7]. Albeit the prevalence of obesity has increased among individuals with type 1 diabetes [8], studies concerning HF in this population are scarce. A cohort study including individuals with type 1 diabetes, showed that poor glycemic control and impaired renal function significantly increased the risk of HF [9]. A publication from our group has also shown that obesity is causally related to diabetic nephropathy (DN) [10], which increases the risk of HF several-fold besides increasing all-cause mortality in type 1 diabetes [9, 11]. However, the impact of obesity on the risk of HF in individuals with type 1 diabetes, especially at different stages of DN, is still unknown. Beyond that, previous studies regarding obesity in type 1 diabetes have used body mass index (BMI) for the obesity definition [8, 10] which has limitations [12]. BMI does not reflect body fat distribution, especially central obesity, which has been associated with HF in the general population and people with type 2 diabetes [7, 13–15] beyond an increased risk of all-cause mortality [16]. Furthermore, BMI is not a good estimator of visceral adipose tissue compared to the waist-height ratio (WHtR) in adults with type 1 diabetes, according to a previous publication from our group [17]. Therefore, considering the increasing number of individuals with type 1 diabetes and obesity, understanding the impact of obesity and body fat distribution on the risk of HF in this population is important, since these individuals already have one of the major risk factors for HF, namely living with diabetes for years. Moreover, finding an easy tool for the identification of high-risk individuals in this population is crucial and may help to diagnose those at risk of HF at an early stage and could also serve as a call for action to avoid the grim consequences of HF.

Given that diabetes and obesity are risk factors for HF [2, 7, 9] and that both are also risk factors for DN [9–11], which in itself is an important risk factor for HF [2, 9, 11], we investigated in the present study the association between body fat distribution and the risk of hospitalization or death due to HF, in adults with type 1 diabetes at different stages of DN. Furthermore, we ranked the impact on the outcome of WHtR, a hallmark of central obesity [18, 19], among other risk factors for HF, seeking to find an easy and accessible tool for screening individuals at high risk.

## Methods

### Study design

In this observational longitudinal study, the outcome was defined as a composite outcome represented by the first event of HF hospitalization after the baseline visit, or death, due to HF identified from the Finnish Care Register for Health Care until the end of 2017. The primary aim was to investigate the association between body fat distribution, classified as central obesity and general obesity, and the outcome in adults with type 1 diabetes during a follow-up period of 16.4 years (12.4–18.5). Then, using the z statistics, the association between WHtR (a measure of central obesity) and the outcome was compared with the association between BMI (a measure of general obesity) and the outcome. As a secondary aim, we performed the same analyses stratifying the sample by DN stages. Then, using z statistics, we ranked the association between WHtR and the outcome among the other risk factors for HF. Additionally, considering a WHtR threshold of 0.5 and a BMI threshold of 30 kg/m^2^, further analyses by DN stages were performed comparing individuals above versus below the threshold regarding the outcome risk. Furthermore, based on our previous study showing that the cardiovascular mortality of individuals with type 1 diabetes starts to increase at a BMI of 24.8 kg/m^2^ [8], we tested the BMI threshold of  ≥  25 kg/m^2^ besides the threshold of  ≥  30 kg/m^2^ for the outcome risk. Finally, we explored whether other anthropometric measures related to central obesity such as waist-hip ratio (WHR), waist and visceral adiposity index (VAI) were associated with the outcome.

### Study population

The Finnish Diabetic Nephropathy (FinnDiane) Study is a nationwide, prospective, multicenter (93 centers across Finland) study since 1997, that aims to identify risk factors for type 1 diabetes complications and recruitment of new participants is still ongoing. From a total of 5401 adults (≥  18 years) with type 1 diabetes in the FinnDiane cohort, individuals with an event of HF hospitalization before the baseline visit, or with end-stage renal disease (ESRD) or unknown albuminuria status at baseline were excluded from this analysis (n  =  733). Thus, we assessed 4668 individuals with anthropometric measures and clinical data from the baseline visit for the occurrence of the first HF hospitalization event, or death, (ICD-8 4270, 4271, 7824, ICD-9 4280–4289, ICD-10, I50) identified from the Finnish Care Register for Health Care or the Causes of Death Register until the end of 2017. Identification of depression was based on data concerning the purchases of antidepressant medication during one year before or after the baseline visit obtained from the Drug Prescription Register (ATC-codes N06A and N06CA). Medications from these classes that are commonly used for the treatment of painful neuropathy (N06AX21, N06AA09, N06AA10, N06AX16) were excluded. The information regarding daily insulin dose per kilogram of body weight and antihypertensive medications were obtained from the FinnDiane questionnaires at the baseline visit. The baseline visit occurred between the years 1997 and 2017 during which the participants underwent a thorough clinical examination, blood and urine samples were collected and several questionnaires were completed by the participants. The same procedures were repeated at each follow-up visit. Type 1 diabetes was defined as age at onset of diabetes under 40 years and permanent insulin treatment initiated within a year from the diabetes diagnosis. The study protocol followed the principles of the Declaration of Helsinki as revised in 2000 and was approved by the Ethical Committee of Helsinki and Uusimaa Hospital District. Written informed consents were obtained from each FinnDiane participant.

### DN stages

DN stage was based on the individuals’ urinary albumin excretion rate (UAER) in timed overnight or 24 h urine (mg/24 h) collections. Normoalbuminuria was defined as a UAER  <  20 µg/min or  <  30 mg/24 h in at least two out of three consecutive urine samples. Microalbuminuria was defined as UAER  ≥  20 and  <  200 µg/min or  ≥  30 and  <  300 mg/24 h and macroalbuminuria as UAER  ≥  200 µg/min or  ≥  300 mg/24 h. End-stage renal disease (ESRD) was defined as dialysis or kidney transplantation and individuals in this category of DN were not included in this study.

### Body fat distribution

BMI was calculated as total body weight (kilograms) divided by the square of the height (meters). BMI was classified as above normal if it was  ≥  25 kg/m^2^, and as obesity if it was  ≥  30 kg/m^2^. The waist was measured in centimeters by a stretch‐resistant tape at the horizontal plane midway between the superior iliac crest and the lower margin of the lowest rib. A waist below 90 cm for men and below 84 cm for women was considered normal [20]. The hip circumference was measured with the same tape around the widest part over the great trochanters, and WHR was calculated by dividing the waist by the hip circumference. A WHR below 0.9 for men and below 0.85 for women was considered normal [20]. The WHtR was calculated by dividing the waist by the height (centimeters), and values below 0.5 were considered normal for both sexes [18, 19]. VAI was calculated by the formula previously described [21]. General obesity was defined by a BMI  ≥  30 kg/m^2^, whereas central obesity by a WHtR  ≥  0.5.

### Statistical analyses

Data on categorical variables are presented as frequencies, continuous variables as means (±  standard deviation, SD) for normally distributed values and otherwise as medians (interquartile range, IQR). Between-group comparisons were performed with the χ^2^ test for categorical variables, with ANOVA for normally distributed continuous variables and with Mann–Whitney or Kruskal–Wallis test for non-normally distributed continuous variables.

Multivariable Cox-regression analysis was used to assess the impact of central obesity (represented by WHtR) and general obesity (represented by BMI) on the risk of hospitalization or death due to HF adjusted for risk factors [22] such as sex, age at onset of diabetes, duration of diabetes, glycated hemoglobin A1c (HbA_1c_), systolic blood pressure (SBP), high-density lipoprotein cholesterol (HDL-C), triglycerides, smoking, lipid-lowering, antihypertensive and antidepressant medication, estimated glomerular filtration rate (eGFR) and DN stage. Follow-up time was counted from the baseline visit until the outcome occurred up to the end of 2017. First analyses were done in the pooled population, then, subgroup analyses according to DN stages were performed adjusting for the same risk factors described above, except DN stage. The relevance ranking of the association with the outcome was based on z statistics [23]. Given that WHtR was the anthropometric measure most strongly associated with the outcome, we performed a ranking using the z statistics of the impact of WHtR and other risk factors for HF on the outcome. Further, using the well-established normal threshold for each anthropometric measure, except VAI that does not have a threshold of normality, two groups were created to compare the risk of the outcome between individuals above and below the threshold by each DN stage.

Since the interactions between sex and the anthropometric measurements were not significant, the analyses were conducted by pooling men and women together. All analyses were performed with the Statistical Analysis System version 9.4 (SAS Institute, Cary, NC, USA).

## Results

During a median follow-up of 16.4 (IQR 12.4–18.5) years, 323 incident cases of hospitalization or death due to HF occurred. The HF hospitalization incidence was 6.6% (n  =  308), with in-hospital mortality of 11.4% (n  =  35). Further 15 deaths occurred without previous hospitalization. In total, there were 50 deaths among 4668 individuals included in the study. According to the DN stage, 3.4% (n  =  112), 9.6% (n  =  62) and 21.3% (n  =  149) of the incident cases occurred in individuals with normo-, micro- and macroalbuminuria stages, respectively.

At the baseline visit, compared to individuals who did not develop the outcome, those who developed had a longer duration of diabetes, higher BMI, WHR, WHtR, waist and VAI, worse glucose and lipid control, lower eGFR and insulin sensitivity, despite similar blood pressure and distribution of sex (Table 1). The baseline characteristics of all individuals and by the stage of albuminuria (micro- and macroalbuminuria combined) according to the incidence of HF hospitalization are depicted in Table 1.

**Table 1:** Clinical characteristics of participants at baseline according to the incidence of heart failure hospitalization or death and the stage of albuminuria

	All		Normoalbuminuria		Albuminuria	
	HF −	HF +	HF −	HF +	HF −	HF +
n (%)	4345 (93.1)	323 (6.9)	3212 (68.8)	112 (2.4)	1133 (24.3)	211 (4.5)
Sex women (%)	49.7	44.9	52.6	55.4	41.6	39.3
Age (years)	36.6 (27.8–46.6)	48.9 (41.0–56.6)***	35.6 (26.5–45.4)	53.3 (43.7–60.9)***	39.3 (31.4–48.7)	47.5 (40.3–53.4)***
Age at onset of diabetes (years)	14.8 (9.5–24.5)	14.7 (9.5–25.4)	16.7 (10.6–26.1)	20.2 (12.0–32.1)**	11.4 (7.3–17.4)	13.0 (8.3–20.8)*
Duration of diabetes (years)	19.0 (10.4–28.4)	31.4 (24.1–38.1)***	16.0 (8.3–26.7)	29.7 (18.3–39.2)***	26.0 (19.5–33.4)	32.1 (26.2–37.0)***
Height (cm)	171.5 ± 9.3	168.9 ± 9.5***	171.4 ± 9.3	166.8 ± 9.5***	170.8 ± 9.3	170.0 ± 9.3
Weight (kg)	74.1 ± 13.5	75.9 ± 15.7*	73.6 ± 13.1	71.3 ± 13.1	75.5 ± 14.3	78.4 ± 16.4*
BMI (kg/m^2^)	24.8 (22.7–27.1)	25.8 (23.4–29.1)***	24.6 (22.5–26.8)	25.1 (22.9–27.9)	25.4 (23.1–28.0)	26.6 (23.8–29.9)**
WHR	0.86 ± 0.08	0.91 ± 0.09***	0.85 ± 0.08	0.87 ± 0.08*	0.89 ± 0.08	0.93 ± 0.09***
WHtR	0.49 (0.46–0.55)	0.54 (0.49–0.59)***	0.48 (0.45–0.53)	0.51 (0.47–0.56)***	0.51 (0.47–0.56)	0.55 (0.50–0.61)***
Waist (cm)	85.6 (77.0–93.0)	90.0 (81.0–100.0)***	83.0 (76.5–91.0)	87.0 (78.0–93.0)*	88.0 (80.0–96.5)	93.0 (84.0–104.0)***
VAI	1.14 (0.78–1.79)	1.66 (1.05–2.79)***	1.06 (0.74–1.60)	1.31 (0.93–1.96)***	1.46 (0.94–2.48)	1.85 (1.20–3.05)***
HbA_1c_ (%)	8.33 ± 1.45	9.01 ± 1.61***	8.16 ± 1.41	8.39 ± 1.37	8.79 ± 1.47	9.33 ± 1.63***
hs-CRP (mg/L)	1.78 (1.01–3.63)	2.72 (1.54–5.48)***	1.65 (0.95–3.36)	2.31 (1.15–3.66)**	2.21 (1.23–4.36)	2.91 (1.77–6.36)***
Systolic blood pressure (mmHg)	130 (120–142)	143 (130–158)	128 (119–139)	136 (125–151)***	138 (126–152)	148 (135–160)***
Diastolic blood pressure (mmHg)	80 (72–85)	80 (73–88)	79 (71–85)	77 (71–86)	81 (75–88)	81 (76–89)
Total cholesterol (mmol/L)	4.80 (4.21–5.41)	5.11 (4.51–5.85)***	4.71 (4.15–5.30)	4.86 (4.25–5.57)**	5.05 (4.40–5.69)	5.26 (4.66–5.94)**
HDL-cholesterol (mmol/L)	1.33 (1.10–1.60)	1.22 (0.98–1.52)***	1.36 (1.13–1.63)	1.24 (1.03–1.58)*	1.25 (1.01–1.51)	1.19 (0.92–1.47)*
LDL-cholesterol (mmol/L)	2.93 (2.42–3.54)	3.26 (2.61–3.78)***	2.86 (2.39–3.43)	2.97 (2.50–3.66)	3.20 (2.58–3.78)	3.32 (2.79–3.91)*
Triglycerides (mmol/L)	0.99 (0.75–1.41)	1.25 (0.92–1.88)***	0.94 (0.72–1.29)	1.04 (0.79–1.44)**	1.20 (0.88–1.78)	1.37 (1.07–2.09)***
Smoking history (yes)	44.8	56.7***	41.3	48.1	55.0	61.2
eGFR (ml/min/1.73 m^2^)	91.4 ± 25.6	63.0 ± 27.6***	97.6 ± 19.8	78.7 ± 20.0***	73.8 ± 31.5	54.6 ± 27.5***
eGDR (mg/kg/min)	6.70 ± 2.43	4.37 ± 2.11***	7.41 ± 2.19	5.77 ± 2.02***	4.68 ± 1.89	3.61 ± 1.74***
Daily insulin (IU/kg body weight)	0.67 (0.52–0.83)	0.62 (0.49–0.77)*	0.66 (0.52–0.83)	0.59 (0.45–0.75)*	0.67 (0.53–0.84)	0.65 (0.53–0.78)
Antihypertensive medication (%)	32.2	78.1***	14.9	57.1***	81.1	89.4**
ACEi only (%)	12.8	15.2	5.7	8.9	33.2	18.5***
ARB only (%)	2.4	2.2	1.3	2.7	5.5	1.9*
Beta-blocker only (%)	1.9	5.0**	1.9	12.5***	2.0	1.0
Calcium channel blocker only (%)	0.4	2.5***	0.2	4.5***	1.0	1.4
Diuretic only (%)	0.1	1.6***	0	3.6	0.4	0.5
ACE plus other AHM (%)	9.6	32.5***	3.6	14.3***	26.6	42.2***
ARB plus other AHM (%)	3.8	11.8***	1.9	4.5	9.1	15.6*
Beta-blocker plus AHM (%)	6.7	34.1***	2.6	17.9***	18.6	42.7***
Lipid-lowering medication (%)	11.4	31.9***	8.3	23.2***	20.1	36.5***
Antidepressant medication (%)*	7.0	13.6***	6.6	15.2**	8.1	12.8*

Data are shown as percentages for categorical variables, median (interquartile range) for non-normally distributed continuous variables and mean  ±  standard deviation for continuous variables with a normal distribution

HF  +  means individuals who presented the outcome and HF − who did not

HF  +  was compared with HF − in each group (all individuals, normoalbuminuria and albuminuria) using the Chi-squared test, Mann Whitney U test, and independent samples’ t test, respectively

*BMI* body mass index; *WHR* waist-hip ratio; *WHtR* waist-height ratio; *VAI* visceral adiposity index; *HbA*_*1c*_ glycated hemoglobin A_1c_; *HDL-cholesterol* high-density lipoprotein cholesterol; *eGFR* estimated glomerular filtration rate; *eGDR* estimated glucose disposal rate; *ACEi* angiotensin-converting-enzyme inhibitor; *ARB* angiotensin II receptor blocker; *AHM* antihypertensive medication

p value *  <  0.05; **  <  0.001; ***  <  0.0001 refers to the comparison between HF  +  vs HF − in each group

Compared to BMI, the WHtR was strongly associated with the outcome (Table 2), as well as the other anthropometric measures related to central obesity such as WHR [HR 1.46 per 0.1 increase, 95% CI (1.22–1.74), z = 4.212] and waist [HR 1.02 per one cm increase, 95% CI (1.01–1.02), z = 3.174]. However, VAI showed the weakest association with the outcome [HR 1.04 per one unit increase, 95% CI (1.01–1.08), z = 2.712].

**Table 2:** The impact of central obesity (WHtR) versus general obesity (BMI) on the risk of heart failure hospitalization or death

	HR (95% CI)	p value	z value
WHtR (per 0.1)	1.51 (1.26–1.81)	1.11E-05	4.395
WHtR (per 1 SD)	1.30 (1.16–1.46)	1.11E-05	–
BMI (per 1 kg/m^2^)	1.05 (1.01–1.08)	6.65E-03	2.714
BMI (per 1 SD)	1.18 (1.05–1.33)	6.65E-03	–

Multivariable Cox-regression model was adjusted for sex, age at onset of diabetes, duration of diabetes, glycated hemoglobin A_1c_, systolic blood pressure, HDL-cholesterol, triglycerides, history of smoking, lipid-lowering, antihypertensive and antidepressant medications, DN stage and estimated glomerular filtration rate

*HR* hazard ratio; *CI* confidence interval; *SD* standard deviation; *WHtR* waist-height ratio; *BMI* body mass index

In the subgroup analysis by DN stages, WHtR was associated with the outcome at the micro- and macroalbuminuria stages, but not at the normoalbuminuria stage (Table 3). A similar pattern was seen for the other anthropometric measures related to central obesity such as WHR and waist, except VAI (Additional file 1: Table S1). Instead, VAI was associated with the outcome only at the normoalbuminuria stage (Additional file 1: Table S1), whereas BMI was associated with the outcome only at the macroalbuminuria stage (Table 3).

**Table 3:** The impact of central (WHtR) versus general obesity (BMI) on the risk of heart failure hospitalization or death in different stages of diabetic nephropathy

	Normoalbuminuria	Microalbuminuria	Macroalbuminuria
	HR (95% CI)		
n (%)	3324 (71.2)	644 (13.8)	700 (15.0)
WHtR (per 0.1)	1.21 (0.86–1.70)	2.33 (1.48–3.67)	1.64 (1.26–2.13)
WHtR (per 1 SD)	1.08 (0.87–1.35)	1.72 (1.29–2.29)	1.37 (1.16–1.62)
BMI (per 1 kg/m^2^)	1.01 (0.95–1.07)	1.07 (0.99–1.15)	1.07 (1.03–1.12)
BMI (per 1 SD)	0.99 (0.78–1.25)	1.29 (0.97–1.71)	1.28 (1.08–1.52)

Multivariable Cox-regression model was adjusted for sex, age at onset of diabetes, duration of diabetes, glycated hemoglobin A_1c_, systolic blood pressure, HDL-cholesterol, triglycerides, history of smoking, lipid-lowering, antihypertensive and antidepressant medications, and estimated glomerular filtration rate

*HR* hazard ratio; *CI* confidence interval; *SD* standard deviation; *WHtR* waist-height ratio; *BMI* body mass index

According to the z statistics ranking in the total sample, HbA_1c_ was the most relevant modifiable risk factor [HR 1.35, 95% CI (1.24–1.46), z = 7.19] and the WHtR was the third modifiable risk factor [HR 1.51, 95% CI (1.26–1.81), z = 4.40] (Additional file 1: Table S2). In the ranking by DN stages, the WHtR was not associated with the outcome at the normoalbuminuria stage (Additional file 1: Table S2). Nevertheless, it was the most important modifiable risk factor [HR 2.33, 95% CI (1.48–3.67), z = 3.66] at the microalbuminuria stage and the third [HR 1.64, 95%CI (1.26–2.13), z = 3.68] at macroalbuminuria stage (Additional file 1: Table S2).

**Fig. 1:**
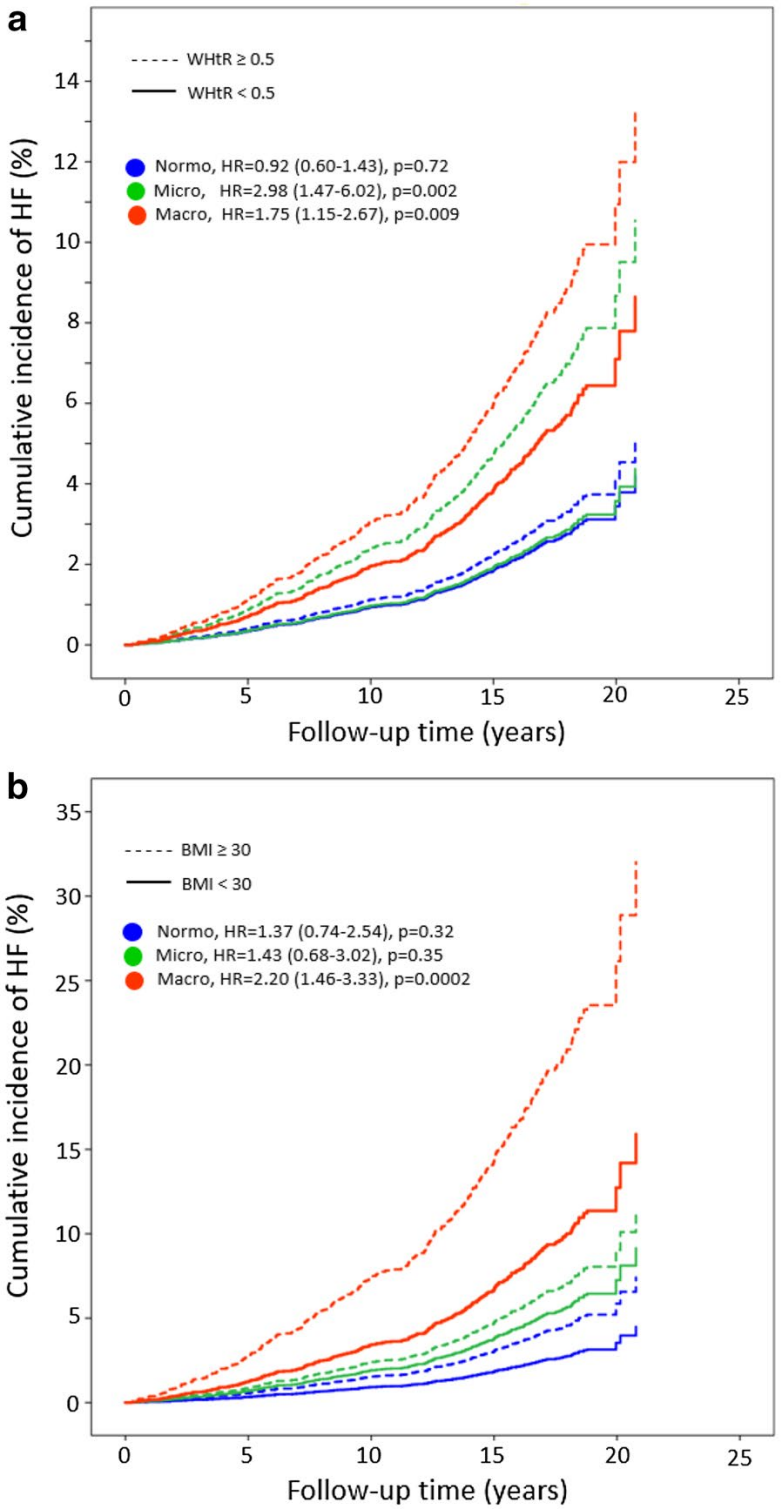
The hazard ratio (HR) of hospitalization or death due to heart failure in individuals above versus below the threshold of central obesity (1a) and general obesity (2a) at different stages of diabetic nephropathy. WHtR, waist-height ratio; central obesity is considered if WHtR  ≥  0.5 for both sexes. BMI, body mass index; general obesity is considered if BMI  ≥  30 kg/m^2^ for both sexes. The multivariable Cox-regression model was adjusted for sex, age at onset of diabetes, duration of diabetes, glycated hemoglobin A_1c_, systolic blood pressure, HDL-cholesterol, triglycerides, smoking, lipid-lowering, antihypertensive and antidepressant medications, and estimated glomerular filtration rate. *HR* hazard ratio. *CI* confidence interval

Comparing individuals above versus below the normal threshold at the microalbuminuria stage, individuals with central obesity (WHtR  ≥  0.5) had a three-fold higher risk (HR 2.98, p  =  0.002) of HF hospitalization or death than those without central obesity (Fig. 1a). Surprisingly, individuals with microalbuminuria and no central obesity (WHtR  <  0.5), had a similar risk as those with normoalbuminuria (Fig. 1a). At the macroalbuminuria stage, central obesity was associated with an almost two-fold increased risk (HR  =  1.75, p  =  0.009) of the outcome (Fig. 1a). Instead, general obesity (BMI of  ≥  30 kg/m^2^) was not associated with the outcome at the normo- and microalbuminuria stages, but only at the macroalbuminuria stage (HR 2.20, p  <  0.0002; Fig. 1b). Interestingly, using a BMI threshold of 25 kg/m^2^, we found that individuals with a BMI  ≥  25 kg/m^2^ showed a 2.5-fold higher risk (HR 2.51, p  =  0.003) of the outcome at the microalbuminuria stage and 1.5-fold higher risk (HR 1.50, p  =  0.04), at the macroalbuminuria stage compared to those below BMI of 25 kg/m^2^ (Additional file 2: Figure S1), which follows the pattern of the association between central obesity and the outcome according to DN stages. The hazard ratio and cumulative incidence of the outcome according to the threshold of waist and WHR are depicted in Additional file 2: Figure S1.

## Discussion

The main finding of the present study is showing that central obesity is strongly associated with the risk of hospitalization or death due to HF in type 1 diabetes and that WHtR, a hallmark of central obesity, is an important modifiable risk factor for this outcome in this population. Furthermore, despite albuminuria being a relevant risk factor for HF per se*,* we showed that individuals with microalbuminuria and a normal WHtR have a similar risk of the outcome as those with normoalbuminuria*.*

In the present study, the incidence of hospitalization due to HF was 6.6%, varying from 3.4 to 21.3% depending on the stage of albuminuria. Our results are in line with previous publications concerning HF hospitalization in type 1 diabetes [9, 24].

Similar to the Swedish cohort studies [9, 24] and the DCCT/EDIC study [25], we found that HbA_1c_ is the most important modifiable risk factor for HF. However, we showed for the first time in type 1 diabetes the impact of central obesity (WHtR  ≥  0.5) versus general obesity (BMI  ≥  30 kg/m^2^) on a hard outcome of HF such as hospitalization or death, according to different stages of DN. Moreover, we showed that WHtR, a hallmark of central obesity, is a relevant and modifiable risk factor among the most important well-known risk factors for HF. In the subgroup analysis by DN stages, the WHtR was not associated with the outcome at the normoalbuminuria stage, although it was strongly associated when albuminuria was present, especially at the microalbuminuria stage. There are two possible reasons why the WHtR was not an important risk factor at the normoalbuminuria stage. First, individuals with type 1 diabetes and normoalbuminuria have a lower percentage of visceral adipose tissue than those with albuminuria [17], which reduces the power of central obesity to predict the outcome. Second, individuals with normoalbuminuria lack an important risk factor for HF, namely the presence of albuminuria [2, 9]. Possibly, central obesity may need the presence of albuminuria to impact the risk of HF hospitalization or death. This rationale would also explain the difference in the outcome risk between the individuals with and without central obesity at the microalbuminuria and the macroalbuminuria stages. The additional risk related to central obesity can also be seen in individuals with microalbuminuria and normal WHtR that have a similar risk as those with normoalbuminuria.

Central obesity and visceral fat have been associated with increased risk of HF in the general population and individuals with type 2 diabetes [7, 13–15]. In the current study, we showed that central obesity, represented by WHtR, was strongly associated with HF hospitalization or death also in individuals with type 1 diabetes. This is in line with our previous research, in which WHtR and waist were the anthropometric measures that best estimated the visceral adipose tissue in adults with type 1 diabetes, far better than BMI [17]. The mechanisms involved in the relationship between central obesity and HF are not clear. However, considering that the visceral adipocytes produce inflammatory cytokines leading to systemic inflammation [26, 27], a systemic pro-inflammatory state, endothelium dysfunction, interstitial fibrosis and cardiomyocyte stiffness have been discussed as possible drivers of myocardial dysfunction [28]. In line with this hypothesis, individuals who had developed the outcome had higher hs-CRP compared to those who did not present the outcome (Table 1).

The hazard ratio of BMI for the outcome in our study was similar to a previous cohort study in type 1 diabetes [24]. Although BMI was associated with the outcome, it was not as good as WHtR. Beyond that, the BMI of 30 kg/m^2^ was able to discriminate high-risk individuals only at the macroalbuminuria stage, which is already an advanced stage of DN with a high risk of HF [9]. On the other hand, using a BMI threshold of 25 kg/m^2^, it was possible to discriminate high-risk individuals similar to the anthropometric measures related to central obesity. This is in line with our previous publication in which the percentage of visceral adipose tissue associated with the BMI of 25 kg/m^2^ was close to the percentage of visceral adipose tissue associated with the WHtR of 0.5, which defines central obesity [17]. Moreover, another publication from our group has shown that the cardiovascular mortality of individuals with type 1 diabetes starts to increase from a BMI of 24.8 kg/m^2^, which probably means that a BMI of 25 kg/m^2^ would be a better threshold for cardiovascular disease and HF risk in individuals with type 1 diabetes.

VAI has been associated with cardiometabolic risk in primary care [21] and with cardiovascular events in individuals with and without previous cardiovascular disease as well as in people with and without type 2 diabetes [29, 30]. However, its association with HF has not been investigated in type 1 diabetes. In the current study, albeit it was associated with the outcome, it showed the weakest association compared to the other anthropometric measures in the whole sample. Interestingly, it was the only measure associated with the outcome at the normoalbuminuria stage. Possibly, triglycerides are driving the association between VAI and the outcome since the VAI formula includes triglycerides concentration, which is an important modifiable risk factor at the normoalbuminuria stage in our analysis. However, VAI was not associated with the outcome at the microalbuminuria and macroalbuminuria stages. Notably, VAI was not a good estimator of visceral adipose tissue when compared to WHtR in adults with type 1 diabetes in a previous analysis of the FinnDiane cohort (data not shown).

This study has some limitations. One of them is the lack of information concerning the stages of HF, hence, we could not evaluate the impact of central obesity on the progression of HF. The classification of HF according to reduced or preserved ejection fraction was also not possible due to a lack of data and we did not either have information regarding the serum natriuretic peptide. Nevertheless, the missing data do not reduce the relevance of our findings since we showed the impact of central obesity on the hard outcome of HF, such as hospitalization or death. Finally, since we studied a homogeneous Caucasian-Finnish population with type 1 diabetes, we cannot exclude, whether ethnicity may have an impact on the results.

On the other side, this study has several strengths. The main strength is the long follow up of a large sample of adults with type 1 diabetes with detailed clinical information. Second, to the best of our knowledge, there is no other study showing the impact of central obesity compared to general obesity and other HF risk factors on a hard outcome of HF in type 1 diabetes. Our results motivate future clinical trials to evaluate whether sodium-glucose cotransporter 2 inhibitors, a well-known anti-diabetic medication that reduces HF hospitalization in type 2 diabetes, should be used in individuals with type 1 diabetes and central obesity. Third, this study highlights the relevance of body fat distribution and not only the total amount of body fat for the complications of diabetes. We showed that a simple measure such as WHtR may help clinicians to identify high-risk patients of HF hospitalization. From a clinical perspective, our results emphasize that treating individuals with type 1 diabetes goes further than achieving good glucose control.

## Conclusions

This study showed that in adults with type 1 diabetes, central obesity has a stronger association than general obesity with the risk of hospitalization or death due to HF. Moreover, the WHtR, a hallmark of central, may be considered in the routine consultations of people with type 1 diabetes for screening high-risk individuals.

## Supplementary Information

**Additional file 1: Table S1.** The impact of the waist, WHR and VAI on the risk of heart failure hospitalization or death in different stages of diabetic nephropathy. **Table S2.** The impact of WHtR and well-known risk factors for heart failure on the risk of hospitalization or death due to heart failure in different stages of diabetic nephropathy. **Table S3.** List of physicians and nurses at each of the FinnDiane centers participating in patient recruitment and characterization.

**Additional file 2: Figure S1.** The hazard ratio (HR) of hospitalization or death due to heart failure in individuals above versus below the normal threshold of WHR (Add. 1a), waist (Add. 1b) and BMI (Add. 1c) at different stages of diabetic nephropathy. WHR, waist-hip ratio; WHR is considered normal if < 0.9 for men and < 0.85 for women. Waist is considered normal if < 94 cm for men and < 80 cm for women. BMI, body mass index; BMI is considered normal if < 25 kg/m^2^ for both sexes. The multivariable Cox-regression model was adjusted for sex, age at onset of diabetes, duration of diabetes, glycated hemoglobin A_1c_, systolic blood pressure, HDL-cholesterol, triglycerides, smoking, lipid-lowering, antihypertensive and antidepressant medications, and estimated glomerular filtration rate.

## Data Availability

No data are available. The ethical statement and the informed consent do not allow for free data availability.

## Abbreviations

BMI: Body mass index
eGFR: Estimated glomerular filtration rate
HbA_1c_: Glycated hemoglobin A_1c_
HF: Heart failure
UAER: Urinary albumin excretion rate
VAI: Visceral adiposity index
WHR: Waist-hip ratio
WHtR: Waist-height ratio
