# Supplementary material for: The impact of central obesity on the risk of hospitalization or death due to heart failure in type 1 diabetes: a 16-year cohort study
Source: Cardiovasc Diabetol. 2021 Jul 27;20:153. doi: 10.1186/s12933-021-01340-4 (PMC8314504; doi:10.1186/s12933-021-01340-4)
Supplement: Supplementary file 1 — Additional file 1: Table S1. The impact of the waist, WHR and VAI on the risk of heart failure hospitalization or death in different stages of diabetic nephropathy. Table S2. The impact of WHtR and well-known risk factors for heart failure on the risk of hospitalization or death due to heart failure in different stages of diabetic nephropathy. Table S3. List of physicians and nurses at each of the FinnDiane centers participating in patient recruitment and characterization. [file 12933_2021_1340_MOESM1_ESM.docx]

**The impact of central obesity on the risk of hospitalization or death due to heart failure in type 1 diabetes: a 16-year cohort study**

Erika B Parente, MD, PhD^1,2,3^, Valma Harjutsalo, PhD^1,2,3,4^, Carol Forsblom, PhD^1,2,3^, Per-Henrik Groop, MD, DMSc, FRCPE ^1,2,3,5^, on behalf of the FinnDiane Study Group.

Additional Files

Additional Table 1. The impact of the waist, WHR and VAI on the risk of heart failure hospitalization or death in different stages of diabetic nephropathy.

|  |  | HR (95% CI) |  |
| --- | --- | --- | --- |
|  | Normoalbuminuria | Microalbuminuria | Macroalbuminuria |
| n (%) | 3324 (71.2) | 644 (13.8) | 700 (15.0) |
| WHR (per 0.1) | 1.07 (0.78-1.47) | 2.78 (1.74-4.44) | 1.64 (1.27-2.10) |
| Waist (per 1 cm) | 1.00 (0.98-1.02) | 1.05 (1.02-1.07) | 1.03 (1.01-1.04) |
| VAI (per 1 unit) | 1.16 (1.07-1.25) | 1.11 (0.97-1.26) | 1.02 (0.98-1.06) |

Multivariable Cox-regression model was adjusted for sex, age at onset of diabetes, duration of diabetes, glycated hemoglobin A_1c_, systolic blood pressure, HDL-cholesterol, triglycerides, history of smoking, lipid-lowering, antihypertensive and antidepressant medications, and estimated glomerular filtration rate. HR, hazard ratio; CI, confidence interval; WHR, waist-hip ratio; VAI, visceral adiposity index.

Additional Table 2. The impact of WHtR and well-known risk factors for heart failure on the risk of hospitalization or death due to heart failure in different stages of diabetic nephropathy.

|  | HR (CI 95%) | p-value | z-value |
| --- | --- | --- | --- |
| All |  |  |  |
| Duration of diabetes (year) | 1.09 (1.08-1.11) | <0.0001 | 11.978 |
| Age at onset diabetes (year) | 1.06 (1.04-1.07) | <0.0001 | 8.1973 |
| HbA1c (%) | 1.35 (1.24-1.46) | <0.0001 | 7.1850 |
| eGFR (ml/min/1.73 m^2^) | 0.99 (0.98-0.99) | <0.0001 | -5.4886 |
| WHtR | 1.51 (1.26-1.81) | <0.0001 | 4.3954 |
| Smoking (yes) | 1.73 (1.29-2.31) | 0.0003 | 3.6578 |
| Antihypertensive medication (yes) | 1.85 (1.32-2.60) | 0.0004 | 3.5664 |
| Albuminuria (yes) | 1.44 (1.05-1.98) | 0.025 | 2.2409 |
| Lipid-lowering medication (yes) | 1.32 (1.01-1.72) | 0.045 | 2.0012 |
| Sex (men) | 0.82 (0.64-1.05) | 0.12 | -1.5465 |
| HDL-C (mmol/L) | 0.81 (0.58-1.14)  -1.27) | 0.22 | -1.2192 |
| Systolic blood pressure (mmHg) | 1.00 (1.00-1.01) | 0.21 | 1.0032 |
| Antidepressant medication (yes) | 1.18 (0.84-1.67) | 0.34 | 0.9630 |
| Triglycerides (mmol/L) | 1.06 (0.93-1.20) | 0.39 | 0.8588 |
| Normoalbuminuria |  |  |  |
| Duration of diabetes (year) | 1.10 (1.07-1.13) | <0.0001 | 7.3681 |
| Age at onset diabetes (year) | 1.07 (1.05-1.10) | <0.0001 | 5.5177 |
| Antihypertensive medication (yes) | 3.37 (2.13-5.34) | <0.0001 | 5.1844 |
| Triglycerides (mmol/L) | 1.28 (1.03-1.58) | 0.03 | 2.2137 |
| eGFR (ml/min/1.73 m^2^) | 0.99 (0.97-1.00) | 0.04 | -2.0552 |
| Smoking (yes) | 1.72 (1.02-2.92) | 0.04 | 2.0189 |
| HDL-C (mmol/L) | 0.56 (0.32-1.00) | 0.05 | -1.9485 |
| HbA1c (%) | 1.14 (0.97-1.34) | 0.11 | 1.5882 |
| Systolic blood pressure (mmHg) | 0.99 (0.98-1.00) | 0.16 | -1.4177 |
| Sex (men) | 0.81 (0.52-1.27) | 0.36 | -0.9141 |
| WHtR | 1.13(0.80-1.60) | 0.13 | 0.6797 |
| Lipid-lowering medication (yes) | 1.07 (0.64-1.80) | 0.79 | 0.2712 |
| Antidepressant medication (yes) | 0.97 (0.52-1.82) | 0.93 | -0.0938 |
| Microalbuminuria |  |  |  |
| Duration of diabetes (year) | 1.13 (1.09-1.18) | <0.0001 | 6.6220 |
| WHtR | 2.33 (1.48-3.67) | 0.0003 | 3.6581 |
| HbA1c (%) | 1.37 (1.14-1.65) | 0.0008 | 3.3604 |
| Age at onset diabetes (year) | 1.05 (1.01-1.09) | 0.0065 | 2.7227 |
| Smoking (yes) | 1.97 (1.02-3.84) | 0.04 | 2.0064 |
| Antidepressant medication (yes) | 1.81 (0.84-3.91) | 0.13 | 1.5172 |
| Lipid-lowering medication (yes) | 1.30 (0.68-2.50) | 0.43 | 0.7955 |
| Systolic blood pressure (mmHg) | 1.00 (0.98-1.01) | 0.47 | -0.7184 |
| HDL-C (mmol/L) | 1.24 (0.57-2.72) | 0.63 | 0.5369 |
| Triglycerides (mmol/L) | 1.09 (0.77-1.53) | 0.63 | 0.4849 |
| Antihypertensive medication (yes) | 0.90 (0.48-1.69) | 0.74 | -0.3275 |
| eGFR (ml/min/1.73 m^2^) | 1.00 (0.99-1.02) | 0.77 | 0.2947 |
| Sex (men) | 1.01 (0.55-1.83) | 0.98 | 0.0248 |
| Macroalbuminuria |  |  |  |
| HbA1c (%) | 1.45 (1.28-1.63) | <0.0001 | 6.0492 |
| Age at onset diabetes (year) | 1.06 (1.04-1.08) | <0.0001 | 5.3448 |
| Duration of diabetes (year) | 1.06 (1.03-1.09) | <0.0001 | 4.4908 |
| eGFR (ml/min/1.73 m^2^) | 0.99 (0.98-0.99) | <0.0001 | -3.9535 |
| WHtR | 1.64 (1.26-2.13) | 0.0002 | 3.6812 |
| Systolic blood pressure (mmHg) | 1.01 (1.00-1.02) | 0.02 | 2.3249 |
| Lipid-lowering medication (yes) | 1.56 (1.06-2.30) | 0.02 | 2.2512 |
| Smoking (yes) | 1.46 (0.96-2.25) | 0.09 | 1.7105 |
| Sex (men) | 0.79 (0.54-1.15) | 0.22 | -1.2340 |
| Antihypertensive medication (yes) | 0.59 (0.23-1.51) | 0.27 | -1.0991 |
| Antidepressant medication (yes) | 1.28 (0.75-2.16) | 0.37 | 0.9028 |
| HDL-C (mmol/L) | 1.05 (0.62-1.79) | 0.85 | 0.1951 |
| Triglycerides (mmol/L) | 0.99 (0.83-1.19) | 0.94 | -0.0747 |

Multivariable Cox-regression model was used to calculate the hazard ratio for the outcome per 1 unit increase of each variable, except the WHtR which is for 0.1 increase, and for categorical variables that are related to the history of smoking, male sex, usage of antihypertensive, lipid-lowering, and antidepressant medications. HbA1c, glycated hemoglobin A1c; eGFR, estimated glomerular filtration rate; WHtR, waist-height ratio; HR, hazard ratio; CI, confidence interval.

Additional Table 3. List of physicians and nurses at each of the FinnDiane centers participating in patient recruitment and characterization.
