# Supplementary figures and images for: The impact of central obesity on the risk of hospitalization or death due to heart failure in type 1 diabetes: a 16-year cohort study
Source: Cardiovasc Diabetol. 2021 Jul 27;20:153. doi: 10.1186/s12933-021-01340-4 (PMC8314504; doi:10.1186/s12933-021-01340-4)

Add. 1.a

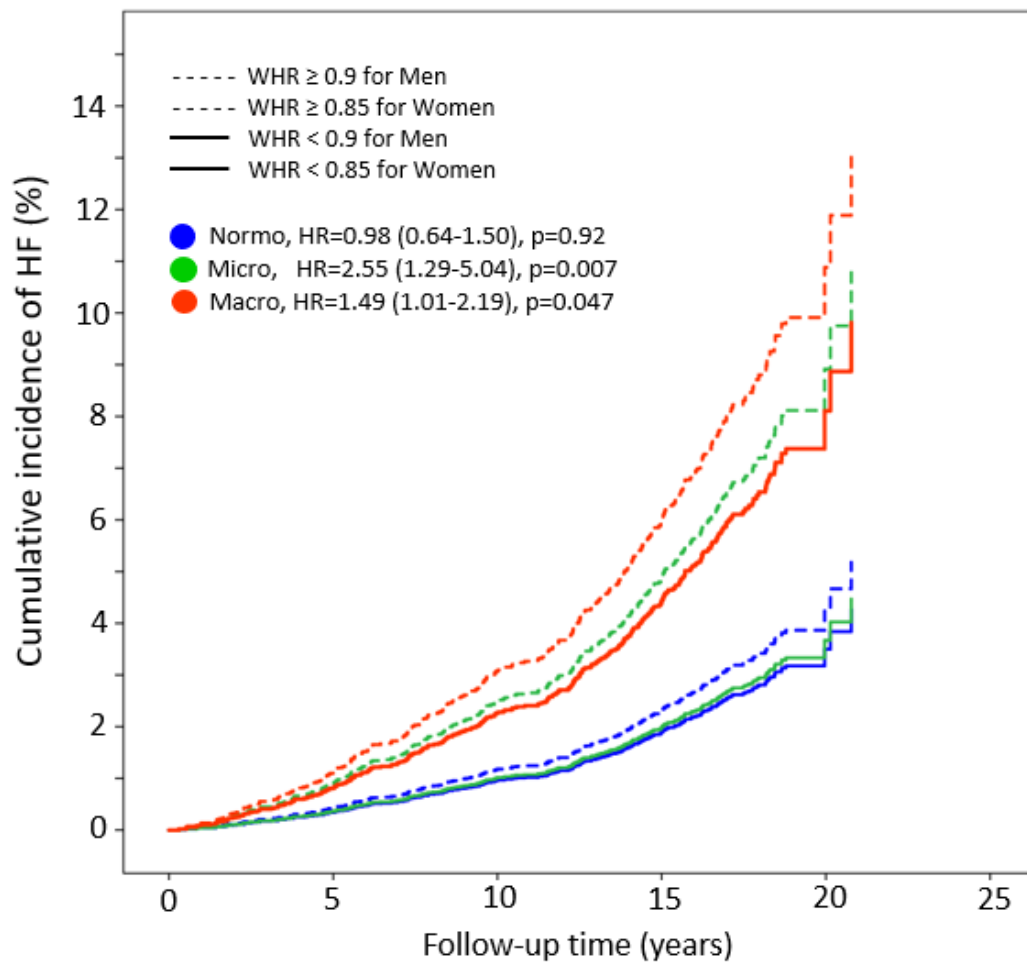

Add. 1.b

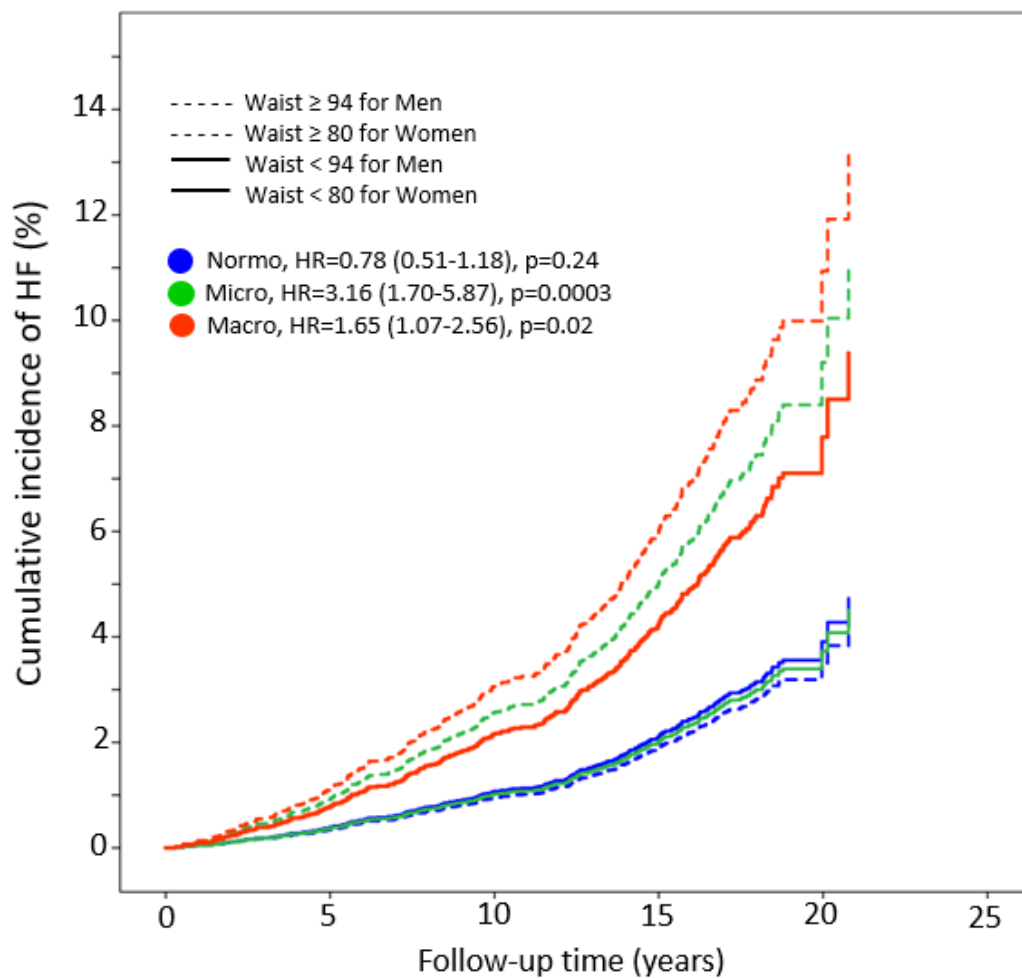

Add. 1.c

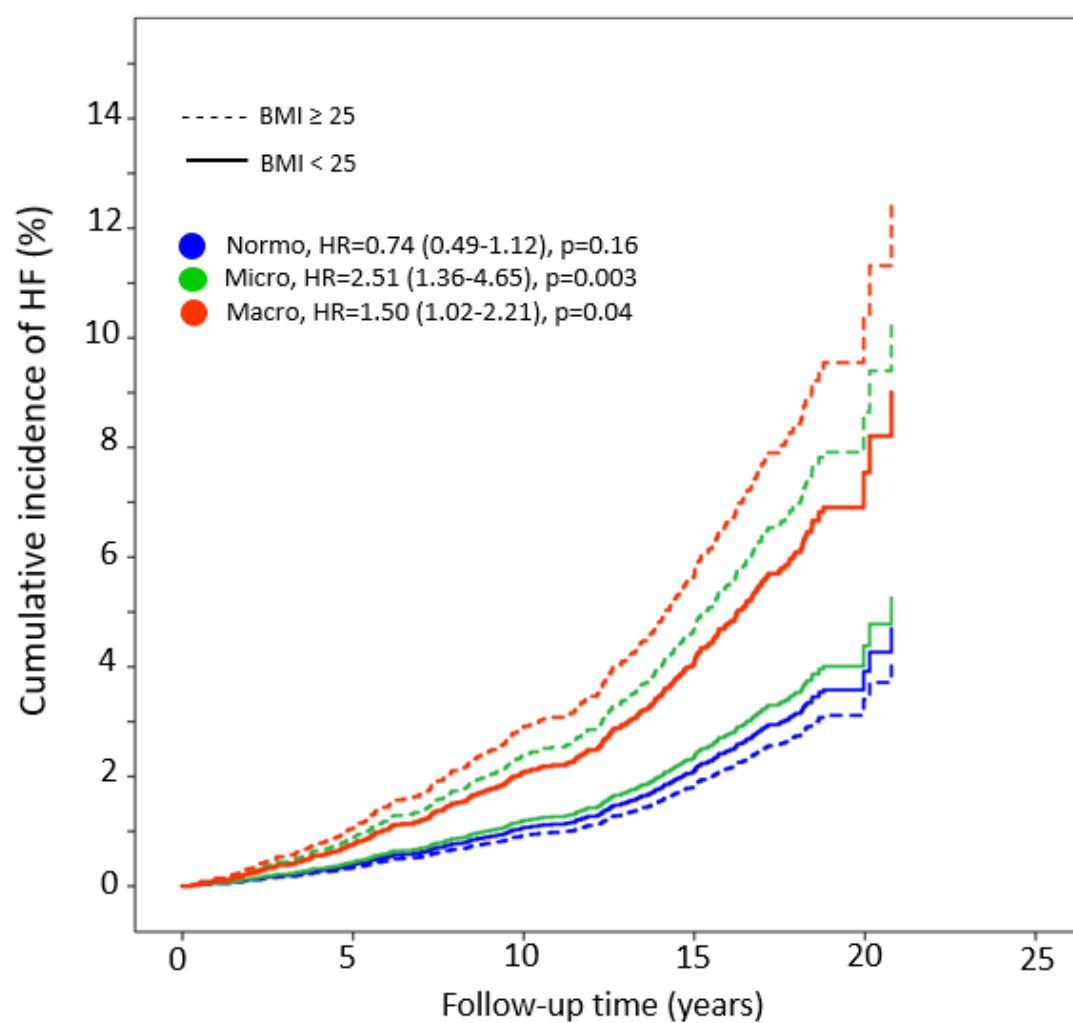

Supplement: Supplementary file 2 — Additional file 2: Figure S1. The hazard ratio (HR) of hospitalization or death due to heart failure in individuals above versus below the normal threshold of WHR (Add. 1a), waist (Add. 1b) and BMI (Add. 1c) at different stages of diabetic nephropathy. WHR, waist-hip ratio; WHR is considered normal if < 0.9 for men and < 0.85 for women. Waist is considered normal if < 94 cm for men and < 80 cm for women. BMI, body mass index; BMI is considered normal if < 25 kg/m2 for both sexes. The multivariable Cox-regression model was adjusted for sex, age at onset of diabetes, duration of diabetes, glycated hemoglobin A1c, systolic blood pressure, HDL-cholesterol, triglycerides, smoking, lipid-lowering, antihypertensive and antidepressant medications, and estimated glomerular filtration rate. [file 12933_2021_1340_MOESM2_ESM.pdf]
